# Application of IgH EtherCAT Master for Ultra-Precision Motion Control of Precision Axes

**DOI:** 10.3390/mi15121483

**Published:** 2024-12-10

**Authors:** Zhihang Pan, Xuesen Zhao, Tianji Xing, Tao Sun

**Affiliations:** Center for Precision Engineering, Harbin Institute of Technology, Harbin 150001, China; zhihangpanhit@163.com (Z.P.); zhaoxuesen@hit.edu.cn (X.Z.); xingtianji2020@foxmail.com (T.X.)

**Keywords:** IgH EtherCAT master, ultra-precision, interpolation algorithm, real-time, motion control

## Abstract

The EtherCAT fieldbus system is widely applied in different types of computerized numerical control (CNC) machine tools due to its outstanding communication performance. In the field of ultra-precision CNC, some machine tools employ controllers that integrate EtherCAT master functionality to achieve real-time communication with other devices; however, the open-source IgH EtherCAT master has rarely been applied to the CNC systems of ultra-precision machine tools. The feasibility of using the IgH EtherCAT master to meet the communication performance requirements of ultra-precision machine tools remains uncertain; therefore, it is necessary to validate the control effect on precision axes under the application of the IgH EtherCAT master. In this work, EtherCAT applications were developed on a personal computer (PC) to alter it to a bus-type controller with the IgH EtherCAT master function. To provide the EtherCAT master with real-time and accurate motion data of the axes, an interpolation algorithm tailored for control experiments was designed, and a G-code data processing method was proposed. Moreover, precision aerostatic linear axes and servo drivers were chosen as EtherCAT slaves for single-axis motion and dual-axis linkage control experiments. The experimental results showed that the motion controller based on IgH can effectively control the precision axes to execute ultra-precision linear and circular interpolation motion.

## 1. Introduction

The machining accuracy of a machine tool is considered a crucial metric, and the communication performance of the fieldbus system has a crucial part in enhancing this accuracy [1,2,3]. A fieldbus is a type of industrial data bus that can be utilized for information transmission between devices in industrial fields. Compared with traditional control systems, a fieldbus not only simplifies the system architecture, but it also improves the reliability of signal transmission. The communication protocols of a fieldbus are open and form some standard bus protocols [4]. With the development of network technology, Ethernet technology has been introduced into the industrial field, and the industrial Ethernet has become a new hotspot for fieldbuses. The industrial Ethernet has additionally improved the information transmission effect in multiple aspects compared with that of the traditional fieldbus [5]; however, in general, the industrial Ethernet can only be used in situations where the real-time requirements are not high. To ensure the industrial Ethernet has high real-time performance, many relevant manufacturers and organizations have proposed various solutions, and the real-time industrial Ethernet has emerged [6]. In 2003, Beckhoff introduced EtherCAT as a novel real-time industrial Ethernet technology, which boasts exceptional communication performances compared to those of other fieldbus solutions, thus attracting significant attention and widespread adoption within the realm of industrial control [7,8,9,10].

In the last few years, EtherCAT has been increasingly employed in high-end machine tools to enhance the information exchange capability between devices of computerized numerical control (CNC) systems. As the originator of EtherCAT, Beckhoff manufactures multiple EtherCAT fieldbus motion controllers that use the TwinCAT to implement the EtherCAT master function [11,12]. Other commercial motion controller brands commonly used in ultra-precision machine tools have also introduced EtherCAT communication capabilities to cater to the needs of machine tools; however, the software programs used in these commercial products to enable EtherCAT communication are not open source, and researchers can only develop CNC systems for ultra-precision machine tools by procuring these products.

In 2009, Etherlab in Germany released an open-source EtherCAT master stack named “IgH”. In the last decade or so, some studies and applications on IgH have been reported in the available literature. Cerreia et al. [13] investigated the real-time performance of the IgH user space and enhanced the real-time performance of IgH control applications by using the real-time application interface (RTAI) kernel. Park et al. [14] proposed a method to enhance the master–slave synchronization accuracy of EtherCAT. They conducted numerous experiments with IgH as the EtherCAT master, thus demonstrating that the proposed method significantly improved the accuracy of master–slave synchronization in EtherCAT networks. Yi et al. [15] studied the distributed clock performance of the IgH EtherCAT master and slaves under the Linux operating system. Their experimental results showed that the distributed clock was sufficiently reliable for motion control applications requiring a distributed clock. Delgado et al. [16] built a motion control system for mobile robots based on the IgH EtherCAT master. Their focus was only on the research of real-time applications, but the requirements of motion accuracy did not reach the level of ultra-precision. The literature review above showed that considerable research efforts have been focused on the real-time and synchronous aspects of IgH. Furthermore, the application of IgH in multi-axis equipment, such as robots, has been widely reported. However, the effective application of IgH to ultra-precision control of precision axes, and even its potential application to the CNC systems of ultra-precision machine tools, requires additional investigation.

In this study, we designed a simple motion control system based on the application of the IgH EtherCAT master and tested the motion accuracy when the system-controlled precision axes executed linear and circular trajectories. First, the theory of EtherCAT communication was studied, and the EtherCAT applications of motion control were designed using the master application interface provided by IgH. Next, we designed a linear and circular trajectory interpolation algorithm for the dual-axis linkage. Subsequently, we proposed a method of processing and transmitting G-code data during motion control in the system. Finally, experimental equipment was set up for single-axis motion and dual-axis linkage control experiments. We captured the network packets of the EtherCAT protocol during the motion control process to analyze the real-time communication performance. We used instruments including laser interferometers and spectral confocal sensors to measure the motion accuracy of the precision axes. The motion control effect under the application of IgH EtherCAT master was evaluated based on the results of the aforementioned experiments.

## 2. Theory of EtherCAT Application

**Figure 1:**
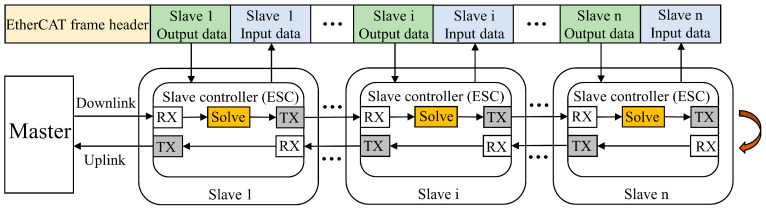
Working principle of EtherCAT.

EtherCAT adopts a master–slave communication architecture to enable data access between devices [17]. In this communication process, the master encapsulates data in a data frame and sends it to the data link. The data frame locates the corresponding slave device using a specific addressing method, and every slave exchanges data with the data frame based on the protocol rules [18]. Upon completion of this process, the data frame returns to the master. Figure 1 illustrates the working principle of EtherCAT.

To achieve motion control, servo drivers are selected as EtherCAT slaves. In order to control the motors to move as required, the EtherCAT master needs to periodically send motion information to the slaves. The exchange of information between the master and slaves is facilitated by using an object dictionary, which represents various parameters. For instance, 607A represents the target position of the motor, and the master can control the motors’ movement by passing position parameters to the driver through 607A. The application uses object words to register process data object (PDO) entries for data exchange during cyclic operation, and the sum of all of the registered PDO entries defines the “process data image”.

**Figure 2:**
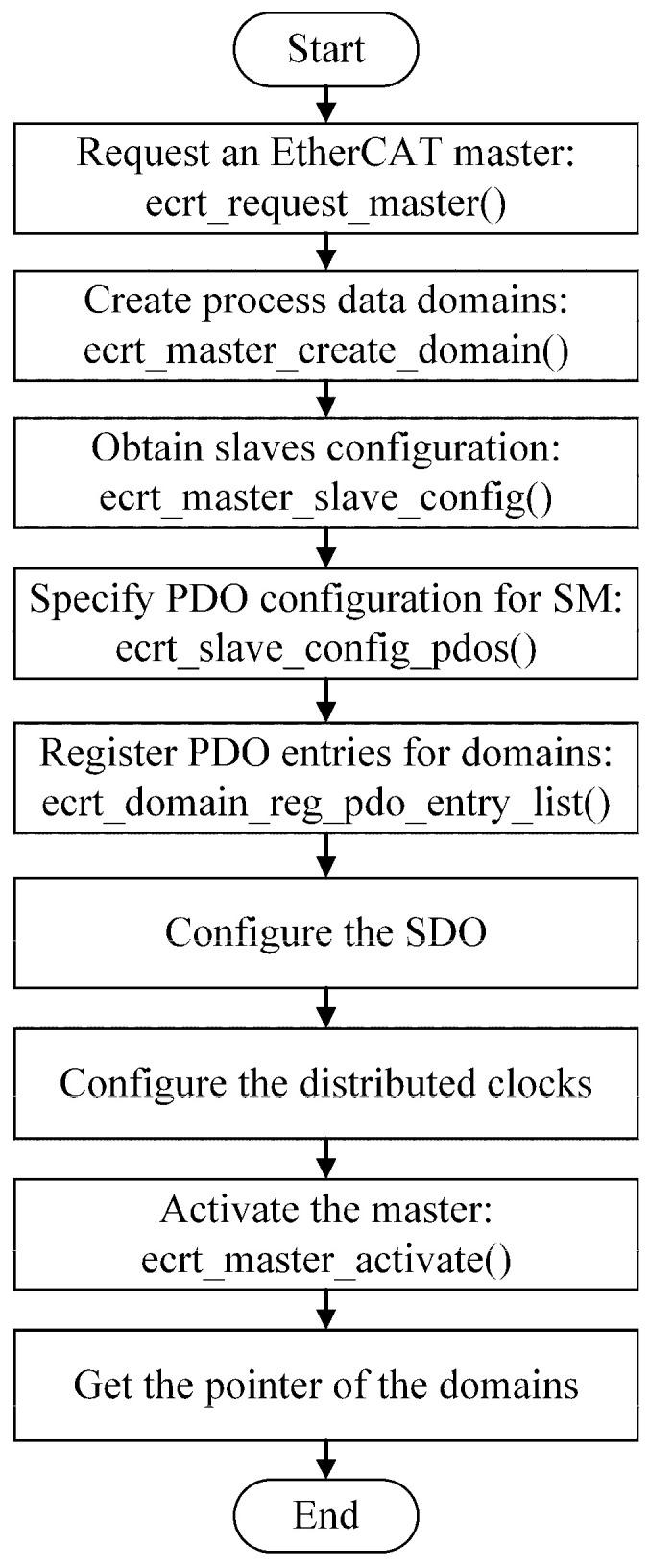
Program flow chart for activating the EtherCAT master.

Before entering cyclic operation mode, the master must be “activated” to calculate the process data image and apply the bus configuration for the first time. In this study, the ActMaster () function is defined to activate the EtherCAT master, and the flowchart of the activation process is shown in Figure 2.

**Figure 3:**
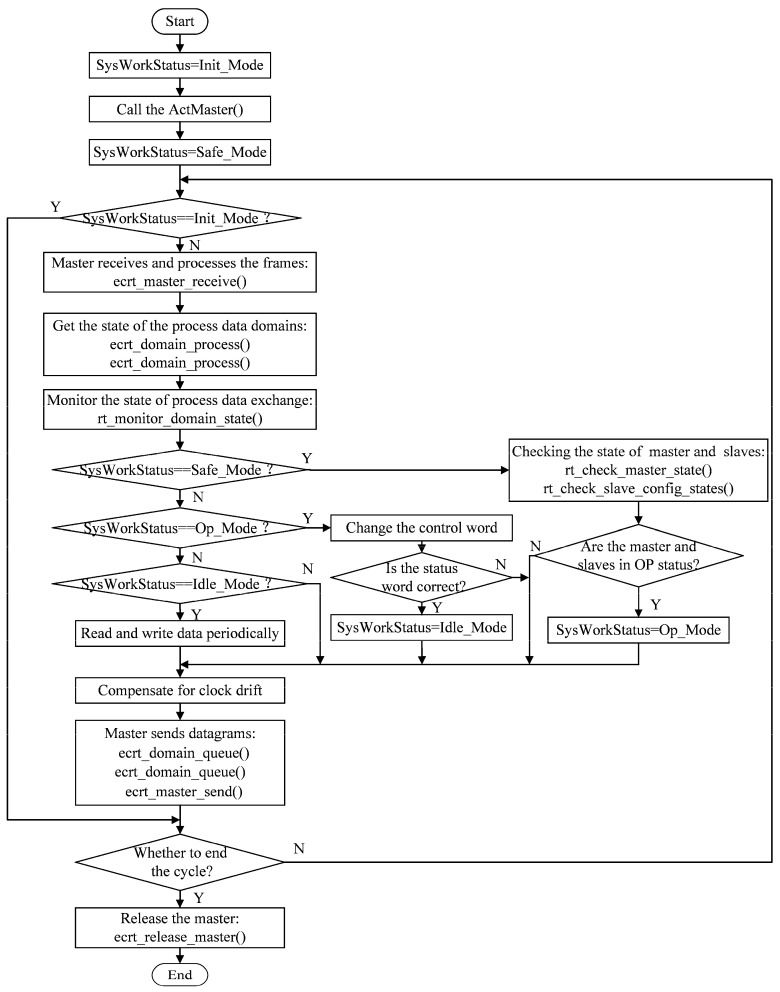
Program flow chart of EtherCAT communication.

The main flow of the EtherCAT communication program is shown in Figure 3. The program sets four working modes for the system: Init_Mode, Safe_Mode, Op_Mode, and Idle_Mode. These modes are switched, based on the state of the master and slaves, and certain operations can only be executed in specific modes.

In the initial stage, the system is set to Init_Mode. After calling ActMaster (), the system mode is converted from Init_Mode to Safe_Mode. In Safe_Mode, the master can continuously receive data frames and process datagrams periodically. In this process, the master checks the process data exchange state of the input and output domains, and then it monitors this state in real time during this process.

In Safe_Mode, the states of the master and slaves are periodically checked. The master is checked for the number of slaves on the bus, the mode of the master’s state machine, and whether at least one Ethernet link is up. The slaves are checked for the online presence and the mode of the state machines. When the state machines of both the master and slaves reach operational mode, the system mode is converted from Safe_Mode to Op_Mode.

In Op_Mode, the control word (6040) of the slaves can be operated on, allowing for operations such as error clearing, power up, start up, and enabling of the drivers and motors. The master then reads the status of the drivers through the status word (6041). If the drivers have reached the set status, the system mode is converted from Op_Mode to Idle_Mode.

In the Idle_Mode state, the system writes the number of pulses to the pointer offset of the output process data domain for the target position, which causes the motors to move the corresponding distance. Additionally, the system can read the pointer offset of the input data domain to obtain the position and speed of the motor. Finally, a function is called to insert the datagram of the input and output domains into the datagram queue of the master and send it to the slaves. During every cycle, the master compensates for the drift of the dynamic clock to ensure synchronization.

## 3. Interpolation Algorithms

Interpolation is a process of inserting a certain number of intermediate points into a theoretical trajectory [19]. The positions of these intermediate points will be transmitted to the drivers via EtherCAT so that the actual trajectory generated by the axes is as close as possible to the theoretical trajectory curve. In this study, an interpolation algorithm is implemented using the principle of data sampling interpolation. The interpolation period is set to 250 µs, which is the same as the EtherCAT communication period.

### 3.1. Algorithm of Linear Interpolation

First, the interpolation algorithm receives G-code information, including the initial position, end position, and feed rate of the linear segment. Based on the start and end coordinates, the total length of the line segment and the total interpolation time can be calculated, respectively, as follows:

(1) $$L=\sqrt{{\left(x_{N}-x_{0}\right)}^{2}+{\left(y_{N}-y_{0}\right)}^{2}},$$

(2) t=L/v,

where *v* is the feed rate. Based on the total time *t* and interpolation period *T*, the line segment is divided into *N* small segments. In most cases, *t* will not be an integer multiple of *T*, so *N* is rounded:

(3) $$N=\left[t/T\right].$$

The undivided portion is typically much smaller than *t*, and thus, this method has a negligible impact on the speed of linear motion. Decomposing the linear trajectory to the *X-* and *Y*-axes, (*x_i_*, *y_i_*) represents the coordinates of the *i*-th interpolation point relative to the start point:

(4) $$\left\{\begin{array}{l} x_{i}=\frac{x_{N}-x_{0}}{N}\cdot i\\ y_{i}=\frac{y_{N}-y_{0}}{N}\cdot i \end{array}\right..$$

### 3.2. Algorithm of Circular Interpolation

The circular arc command in the G-code specifies the start position, end position, feed rate, circular direction, and either the circular radius or the relative coordinate of the circular center to the start point. The circular interpolation algorithm must be implemented based on different cases according to G-code commands.

#### 3.2.1. Determining Coordinate of Circular Center

The circular commands in G-code can be divided into two categories: R instructions, which provide the circular radius, and I-J instructions, which provide the position of the circular center relative to the start point coordinates. For I-J instructions, the coordinate of the circular center can be obtained by adding the I-J value to the coordinate of the start point. However, for R instructions, the central coordinate must be considered in three cases.

In the first case, if the X-coordinates of the start and end points are the same, the circular center can be calculated by the two points and radius. In this way, we can obtain the only Y-coordinate and two undetermined X-coordinates. Similarly, in the second case, if the Y-coordinates of the start and end points are the same, the circular center can be calculated in the same way.

In the third case, if the X-coordinates and Y-coordinates of the start and end points are different, the central coordinates of the circular arc can be calculated using the solution equation method:

(5) $$\left\{\begin{array}{l} (x_{0}-x_{C}{)}^{2}+(y_{0}-y_{C}{)}^{2}=R^{2}\\ (x_{N}-x_{C}{)}^{2}+(y_{N}-y_{C}{)}^{2}=R^{2} \end{array}\right.,$$

where $\left(x_{C},y_{C}\right)$ represents the central coordinates of the circular arc, and $\left(x_{0},y_{0}\right)$ and $\left(x_{N},y_{N}\right)$ are the start and end coordinates of the circular arc. $y_{C}$ in Equation (5) can be expressed as follows:

(6) $$y_{C}=\frac{x_{0}^{2}-x_{N}^{2}+y_{0}^{2}-y_{N}^{2}}{2(y_{0}-y_{N})}-\frac{x_{0}-x_{N}}{y_{0}-y_{N}}.$$

By letting $k=\frac{x_{0}^{2}-x_{N}^{2}+y_{0}^{2}-y_{N}^{2}}{2(y_{0}-y_{N})}$ and $b=-\frac{x_{0}-x_{N}}{y_{0}-y_{N}}$, Equation (5) can be expressed as

(7) $$(1+b^{2})x_{C}^{2}-2[x_{0}+b(y_{0}-k)]x_{C}+x_{0}^{2}+{\left(x_{0}-k\right)}^{2}-R^{2}=0.$$

By letting $A=1+b^{2}$, $B=-2[x_{0}+b(y_{0}-k)]$, and $C=x_{0}^{2}+{\left(x_{0}-k\right)}^{2}-R^{2}$, $x_{C}$ can be calculated from the formula

(8) $$x_{C}=\frac{-B\pm \sqrt{B^{2}-4AC}}{2A}.$$

The results of the calculation are two undetermined central coordinates. The unique $\left(x_{C},y_{C}\right)$ can be determined based on the start and end points, the circular direction, and whether it is a major arc or minor arc.

#### 3.2.2. Determining Angle Corresponding to Point on Circular Arc

**Figure 4:**
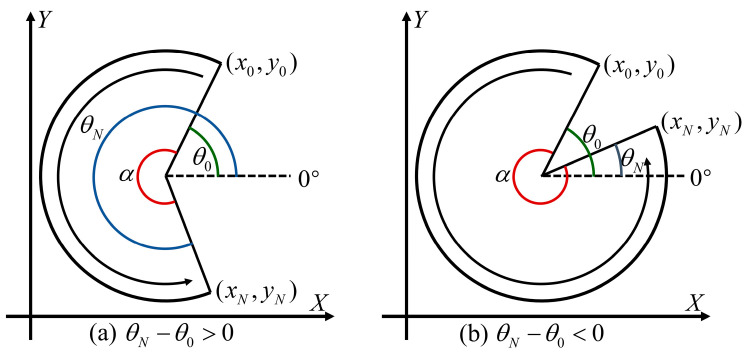
Principle of calculating the radians of a counterclockwise circular arc.

**Figure 5:**
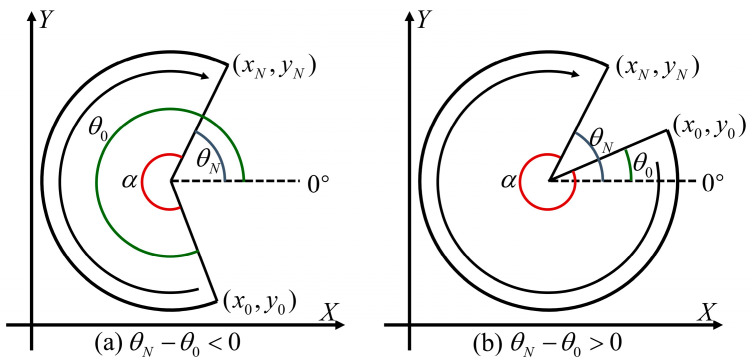
Principle of calculating the radians of a clockwise circular arc.

As shown in Figure 4 and Figure 5, we define a line made from the circular center to the positive direction of *X*-axis as the “zero degree line”. The angle corresponding to the point of the circular arc is defined as the angle between the line connecting the circular center and the point on the circular arc and the “zero degree line”. This angle can be calculated from the coordinates of the point and the center. By using this method, the angle corresponding to any point on the circular arc can be determined, and the angle between any two points can be calculated as well.

The radians of a circular arc are determined by the angle between the start point and the end point on the arc, but these radians cannot be obtained by simple subtraction. Let $\left(x_{0},y_{0}\right)$ be the angle corresponding to the start point, $\left(x_{N},y_{N}\right)$ be the angle corresponding to the end point, and α be the radians of the circular arc. As shown in Figure 4, when the direction of the circular arc is counterclockwise, if $\theta_{N}-\theta_{0}>0$, then $\alpha =\theta_{N}-\theta_{0}$; if $\theta_{N}-\theta_{0}<0$, then $\alpha =2\pi +(\theta_{N}-\theta_{0})$. As shown in Figure 5, when the direction of the circular arc is clockwise, if $\theta_{N}-\theta_{0}<0$, then $\alpha =\theta_{0}-\theta_{N}$; if $\theta_{N}-\theta_{0}>0$, then $\alpha =2\pi -(\theta_{N}-\theta_{0})$.

#### 3.2.3. Circular Interpolation

Based on the angle α, radius R, and feed rate v, the circular arc length L and total interpolation time t can be expressed as

(9) L=α⋅R,

(10) t=L/v.

Based on t and the interpolation period T, the circular arc is divided into N small segments. Similar to line imputation, N needs to be rounded:

(11) $$N=\left[t/T\right].$$

From the start point to the end point, the circular arc is divided into circular segments 1 − N. The angle corresponding to the end point of the *i*-th circular segment can be expressed as

(12) $$\theta_{i}=\theta_{0}+\frac{i}{N}\times \alpha .$$

The coordinates of the *i*-th point can be expressed as

(13) $$\left\{\begin{array}{l} x_{i}=x_{C}+R\times \cos\theta_{i}\\ y_{i}=y_{C}+R\times \sin\theta_{i} \end{array}\right.,$$

where $\theta_{0}$ is the angle corresponding to the start point of the circular arc.

The position of every interpolation point is converted by the master controller into the corresponding number of pulses and transmitted via EtherCAT to the motion axes, which move in unison to execute the circular trajectory specified in the G-code command. The theoretical error generated by this circular interpolation algorithm is less than nanometer level for the ultra-precision machining process parameters commonly used, thereby satisfying the accuracy requirements of the experiments.

When the G-code instruction is an I-J instruction, the circular radius can be calculated based on the coordinates of the start point and the I-J value. The circular interpolation function of the R instruction is then called to execute the interpolation operation.

## 4. Method of G-Code Data Processing

Before installing the IgH EtherCAT master on the PC, a real-time kernel needs to be installed to ensure real-time EtherCAT communication [20,21,22]. In this study, Xenomai was selected as the real-time kernel to handle real-time tasks, while non-real-time tasks were still handled by Linux.

**Figure 6:**
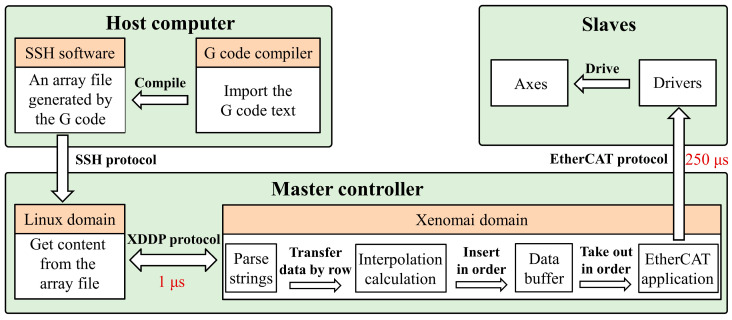
Data processing of G-code in motion control system.

The G-code data processing flow during motion control is shown in Figure 6. The system input is a G-code file, which is compiled by the host computer to avoid overloading the computing tasks in the master controller. The compiled file, called the “array file”, is transmitted to the master controller via the SSH protocol. In the Linux domain, the master controller reads the data from the “array file” and transmits it as a string to the Xenomai domain via the cross-domain datagram protocol (XDDP) [23]. This method of data transmission will not corrupt the real-time environment provided by Xenomai. The cycle time of the XDDP communication program is set to 1 μs to make the transfer much faster than the speed of EtherCAT communication. In the Xenomai domain, strings are parsed as data that will be transmitted to the interpolation application.

**Figure 7:**
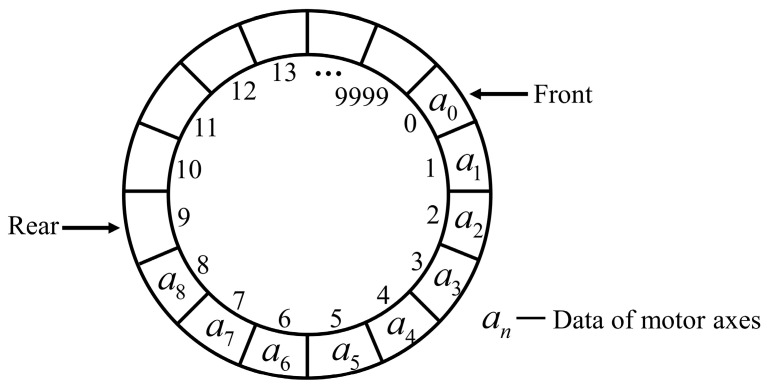
Theoretical structure diagram of ring buffer.

The interpolation program generates data much faster than it is consumed, necessitating the use of a buffer to temporarily store the excess data. To ensure that the data are processed in a first-in-first-out order and to address the issue of a large data volume, a circular queue is applied. As shown in Figure 7, the ring buffer comprises 10,000 data spaces, with every space capable of storing data for one EtherCAT cycle. Although the buffer occupies less than 2 MB of space, it can store a large amount of data, thereby reducing the performance requirements of the computer hardware and resulting in lower costs. The buffer can be in one of three states: empty, normal, or full. Before data are produced by the interpolation task and consumed by the EtherCAT task, the buffer’s state must be determined. In this study, two semaphores are used to coordinate the two tasks based on the buffer’s state.

This study utilized the real-time task management interface provided by Xenomai to establish two real-time tasks for the interpolation program and the EtherCAT program [24]. The EtherCAT task was assigned the highest priority of 99 to ensure stable periodic communication, while the interpolation task was set to the next highest priority of 98 to enable high-speed interpolation. In the event that other non-real-time tasks with lower priority came into conflict with the high-priority tasks, they were required to wait for the high-priority tasks to release the required resources before running.

The interpolation task and the EtherCAT task ran synchronously, with both tasks accessing the shared data in the buffer. However, concurrent access to the buffer by both tasks would lead to data conflicts. To address this issue, we utilized mutex locks to ensure that the two tasks operated on the buffer data sequentially.

## 5. Experimental Verification

### 5.1. Design of Experiments

#### 5.1.1. Experimental Setup

**Figure 8:**
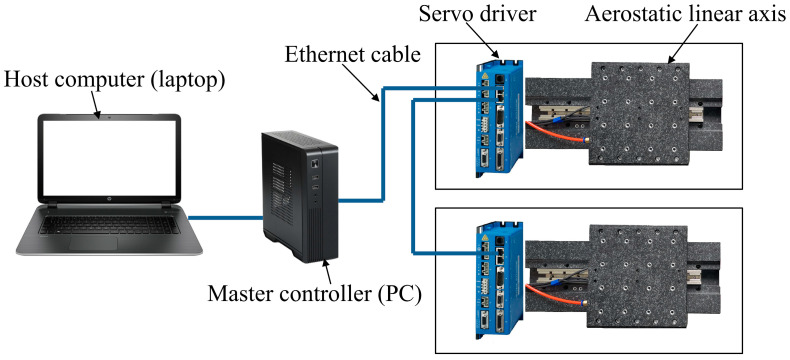
Devices of the system.

The master controller was a PC with two network ports and a four-core 3.1 GHz CPU, running the Ubuntu 16.04 operating system, version 4.9.90 of the Linux kernel, version 3.1 of the Xenomai kernel, and IgH EtherCAT master. The host computer was a laptop with HitCpeCNC V1.0 software designed to work with the master controller. The controlled axes were two high-precision aerostatic linear axes equipped with Copley Xenus Plus XEL series servo drivers (Copley controls, Canton, MA, USA) supporting EtherCAT communication. The hardware of the system is shown in Figure 8.

#### 5.1.2. Experiments of Single-Axis Motion Control

The accuracy of single-axis motion control can be evaluated through the positioning accuracy and repetitive positioning accuracy. When the positioning accuracy and repetitive positioning accuracy are high, it indicates that the motion control accuracy of the system for the aerostatic axis is also high. However, factors including the accuracy and installation error of the grating and guide can also significantly impact the positioning accuracy and repetitive positioning accuracy. To mitigate the influence of other factors on the experiments, this paper implements error compensation during the measurements of the positioning accuracy and repetitive positioning accuracy.

**Figure 9:**
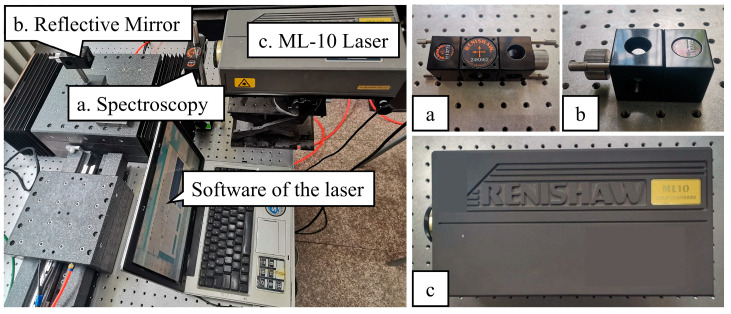
Devices of laser interferometer.

**Figure 10:**
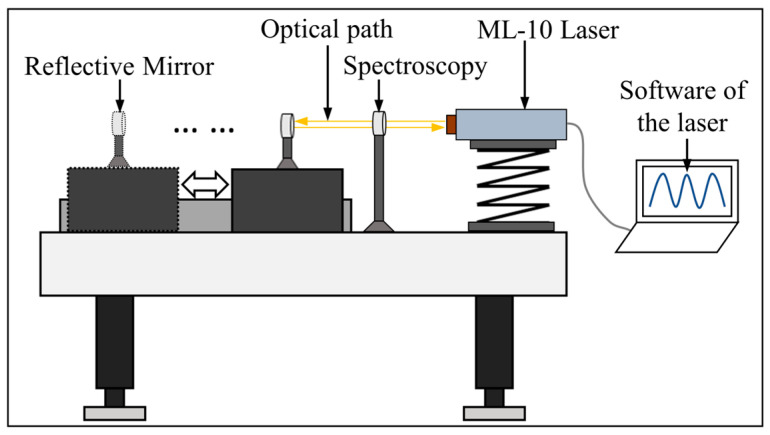
Sketch of the location of single-axis motion control experimental equipment.

**Figure 11:**
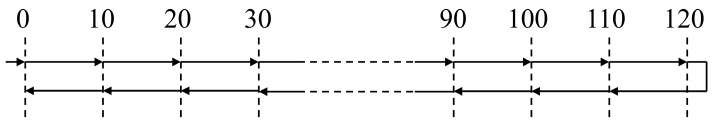
Round trip at first measurement.

The aerostatic linear axis had a stroke of approximately 140 mm, with 120 mm measured in 12 segments, each with a 10 mm distance. The measuring instrument was a Renishaw laser interferometer, as shown in Figure 9. The location of experimental equipment is shown in Figure 10. The measurement included a total of 13 target points with a 2 mm overtravel set at the first and last target points. Because the first measurement aimed to obtain the position deviation for every target point, as shown in Figure 11, only one round-trip measurement was performed.

**Figure 12:**
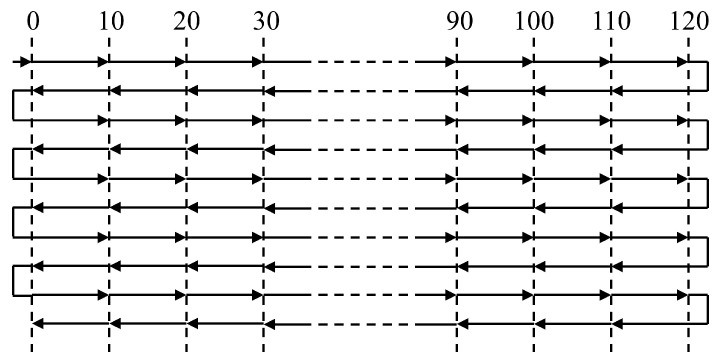
Round trip at the time of error assessment.

The error compensation table was calculated by the laser interferometer based on the positioning error, and the compensation values in the table were used for error compensation in the next measurement. The process of one round-trip measurement and error compensation was repeated multiple times until additional error compensation yielded minimal improvements in the measurement results. Finally, the last measurement was conducted to evaluate the positioning accuracy and repetitive positioning accuracy. In contrast to prior measurements, five round-trip measurements were performed to analyze the results, as shown in Figure 12.

#### 5.1.3. Experiments of Dual-Axis Linkage Control Accuracy

**Figure 13:**
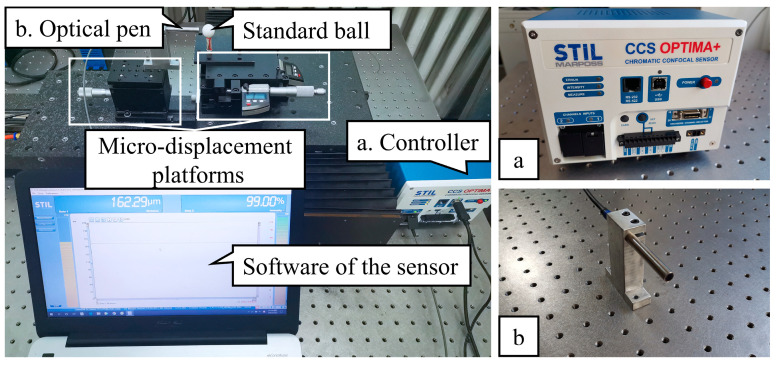
Devices of linkage accuracy testing.

**Figure 14:**
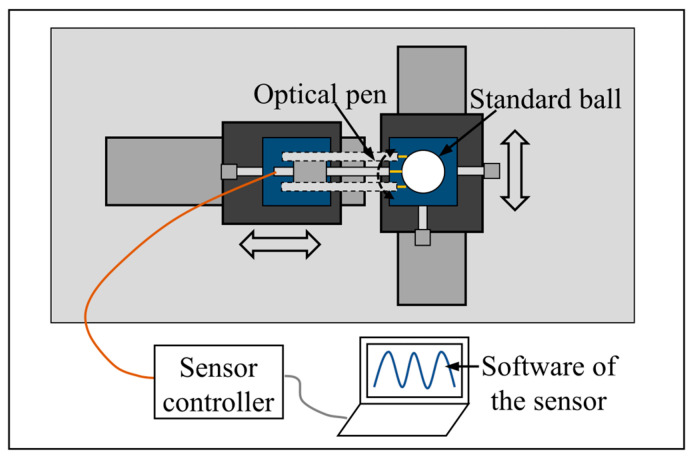
Sketch of the location of linkage accuracy testing experimental equipment.

In this study, the accuracy of the dual-axis linkage was evaluated by measuring the roundness of a standard ball using a spectral confocal sensor and a standard ball with 10 mm radius and 0.3 μm roundness error. As shown in Figure 13, the spectral confocal sensor was equipped with a CCS OPTMA+ controller from STIL and an ENDO 0.2/D8 optical pen with a 4.8 mm working distance and a 220 μm measurement distance. The software of the sensor dynamically displayed the values of the relative displacement between the measured ball and the optical pen probe. The location of experimental equipment is shown in Figure 14. When the probe’s trajectory was the same as the circular arc of the spherical surface, the sensor reading was constant. When the trajectory of the dual-axis interpolation was different from the theoretical circular arc, the sensor reading changed. According to the above principle, we evaluated the accuracy of the dual-axis linkage based on the reading of the sensor.

It was challenging to determine the radius of every latitude line on the surface of the standard ball, which made it impossible to set the radius of the circular interpolation in the G-code. However, the radius of the equatorial circle was known to be 10 mm, so the radius in the G-code was set to 10 mm. Prior to the measurement, the micro-displacement platforms needed to be precisely adjusted based on the sensor indication to ensure that the sensor spot fell on the equatorial circle of the standard ball.

**Figure 15:**
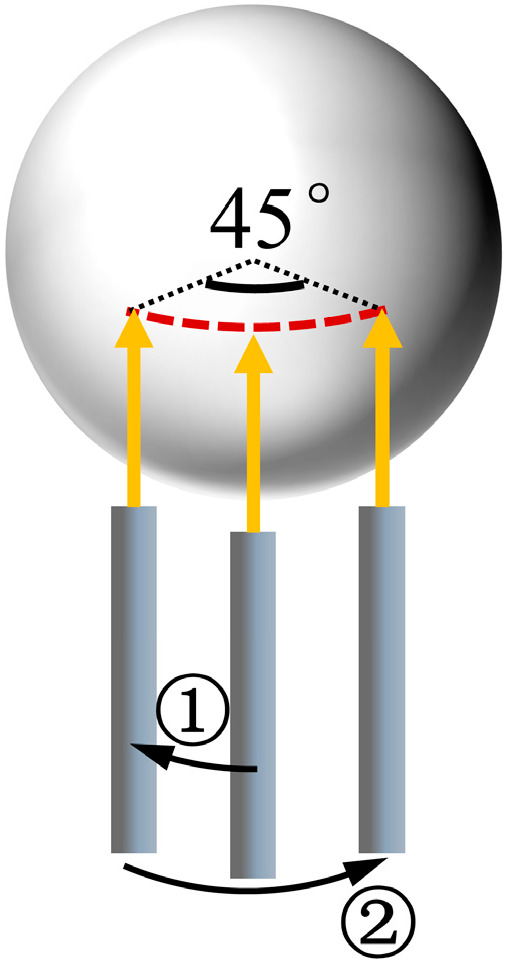
Testing method.

The maximum sampling slope of the sensor could not exceed 23°, so it was important to ensure that the radian of the circular arc swept by the probe was not too large. To address this limitation, we designed the probe to move through a 22.5° circular arc, followed by a 45° circular arc in the reverse direction, as shown in Figure 15. We focused on the data collected during the second circular arc.

#### 5.1.4. Verification Experiment of Linear Interpolation

**Figure 16:**
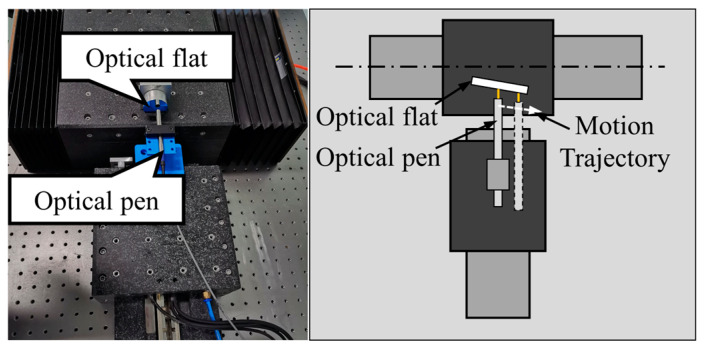
Linear interpolation verification experiment plan.

The experiment on the control accuracy of dual-axis linkage adopted the circular interpolation function. To verify that the proposed method also possesses linear interpolation capabilities, an experimental setup was designed as shown in Figure 16. In this setup, the axis on which the optical flat is placed is defined as the *X*-axis, while the axis where the optical pen is located is defined as the *Y*-axis. A slight angle exists between the optical flat and the *X*-axis, and the optical pen is placed perpendicular to the *X*-axis.

First, the *X*-axis is controlled to move 1 mm to the left, as depicted in the figure. Due to the angle between the optical flat and the motion direction of the *X*-axis, the measurement value of the optical pen increases progressively. The difference between the optical pen’s measurement value at the start and end of the *X*-axis movement is recorded as h mm. Subsequently, the *X*-axis is moved 1 mm to the right to return to its initial position. Finally, the *X*-axis and *Y*-axis are moved simultaneously using the linear interpolation method, with the *X*-axis moving 1 mm and the *Y*-axis moving h mm. During this process, the measurements from the optical pen are used to evaluate the linear interpolation functionality.

#### 5.1.5. Capture of EtherCAT Packets

**Figure 17:**
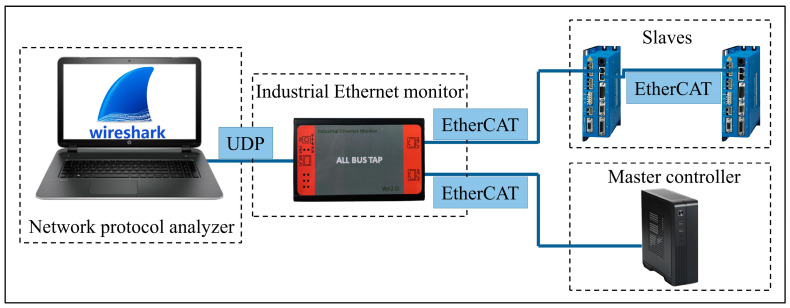
Method of capturing EtherCAT packets.

In order to intuitively evaluate the communication performance of the IgH EtherCAT master, the EtherCAT packets exchanged between the master controller and the slaves were captured and analyzed. The method of capturing EtherCAT packets is shown in Figure 17. An industrial Ethernet monitor, ALLBUS-TAP, was connected to the EtherCAT segment, which could capture EtherCAT frames and record their timestamps accurately. ALLBUS-TAP had three ports, two of which were used for forwarding industrial Ethernet data, and the third was used for uploading the captured data to a PC with the Wireshark software (Version 2.0.2) installed. We captured EtherCAT frames with Wireshark during the motion control experiment and analyzed the communication performance based on them.

### 5.2. Results and Discussion

**Figure 18:**
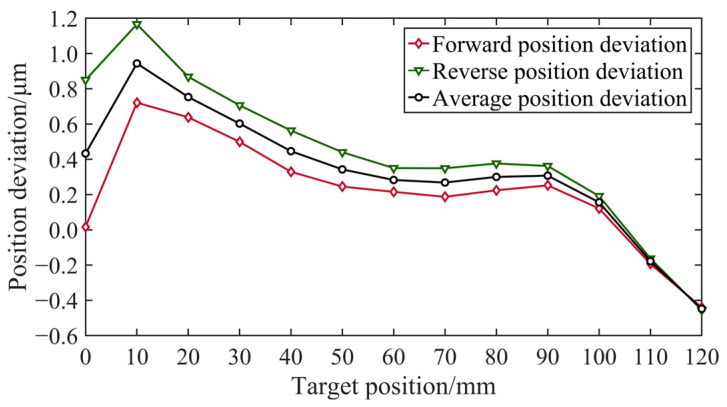
Position deviation of the first measurement.

Figure 18 shows that the maximum position deviation without error compensation was in the micron range. The results of the first measurement indicate significant positional deviations during motion, as well as inconsistencies between the measurements of forward and reverse motions. The causes of these issues, aside from motion control errors, include manufacturing and installation errors of the grating scale, machining and assembly errors of the guide rail, external vibrations, and temperature variations. Among these, random errors such as external vibrations and temperature changes are challenging to control. However, the manufacturing and assembly errors of the grating scale and guide rail can be mitigated using error compensation methods.

To more accurately reflect the impact of motion control on positioning accuracy, a commonly used positioning error compensation method was employed in the experiment. This method involves inversely superimposing positional errors onto the motion commands, thereby reducing positional deviations and backlash caused by other influencing factors. The error compensation table generated from this measurement was utilized for error compensation in the subsequent measurements.

**Figure 19:**
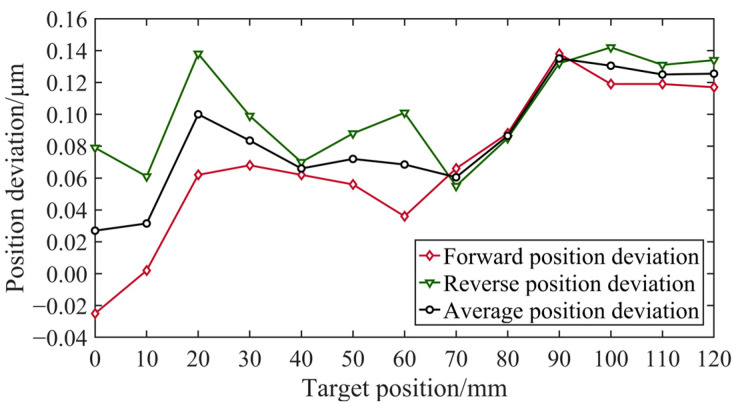
Position deviation of the second measurement.

**Figure 20:**
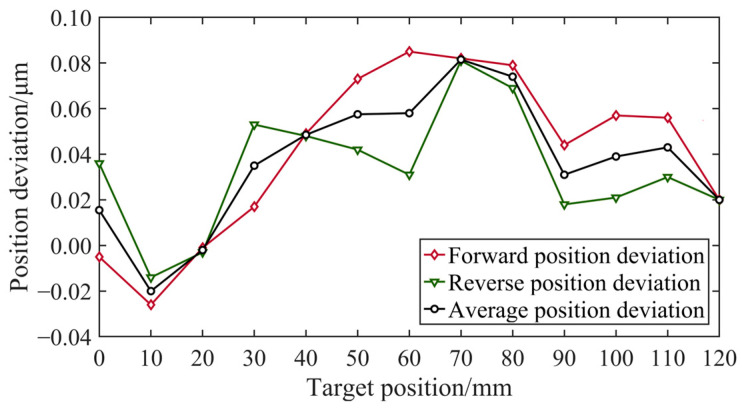
Position deviation of the third measurement.

Figure 19 and Figure 20 present the position deviation results obtained in the second and third measurements, respectively. Table 1 shows that following two rounds of error compensation, the accuracy of the position deviation was significantly improved, with the maximum position deviation reduced from over 1 μm to less than 0.1 μm. It is evident that error compensation effectively minimizes positional deviations caused by factors beyond motion control and significantly reduces discrepancies between forward and reverse motion measurement results. However, the improvement in accuracy from the second to the third measurement was relatively weak. Additional experiments indicated that continuing the error compensation did not lead to significant improvements in the position deviation accuracy. Thus, evaluation experiments for the positioning accuracy and repetitive positioning accuracy were conducted based on the value of the current compensation.

**Figure 21:**
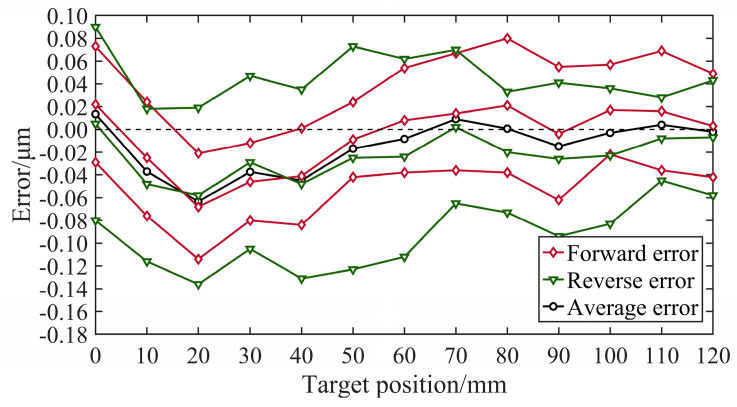
Error analysis.

Figure 21 shows the error analysis generated by the laser interferometer, and Table 2 shows the results of the software analysis for the positioning accuracy. It was found that the positioning accuracy was 0.227 μm and the unidirectional repetitive positioning was 0.196 μm when controlling single-axis movement with the IgH EtherCAT master.

**Figure 22:**
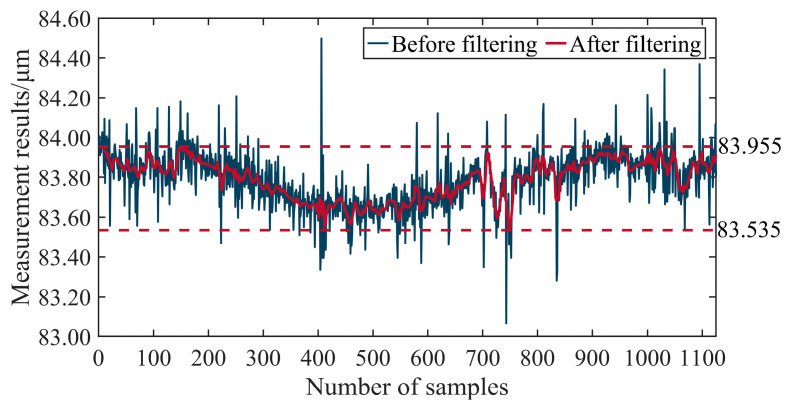
Measurement results for dual-axis linkage.

The results of the dual-axis linkage experiment are presented in Figure 22, showing relatively high noise in the results. The noise may have stemmed from environmental vibrations, motor vibrations, and the surface roughness of the ball. Among these, the surface roughness of the ball typically consists of high-frequency errors at the nanometer scale, while the dual-axis linkage measurement results are at the micrometer to sub-micrometer scale. Therefore, the influence of the ball’s surface roughness on the measurement accuracy can be considered negligible. Upon filtering, the error range was approximately 0.5 μm. Nevertheless, the error encompassed not only the dual-axis linkage control error but also errors from other external factors, such as the shape error of the ball, the installation error of the motion axis, and the error of other measurement devices. Because these extra error factors could not be isolated in the experiments, we could only qualitatively analyze the motion control effect rather than obtaining a precise value of the motion control accuracy.

**Figure 23:**
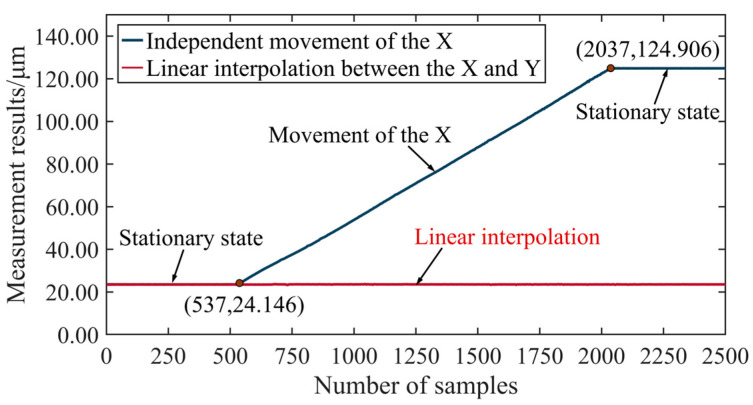
Measurement results for linear interpolation.

The measurement results of the linear interpolation verification experiment are shown in Figure 23. Due to the slight angle between the optical flat and the motion direction of the *X*-axis, the measurement values gradually increase at a constant slope when the *X*-axis moves independently. During the second measurement, the *X*-axis and *Y*-axis perform linear interpolation. As a result, the trajectory of the optical pen relative to the optical flat is parallel to the optical flat, leading to a measurement curve that remains consistently horizontal. This experiment demonstrates that the proposed method in this study effectively supports linear interpolation functionality.

**Figure 24:**
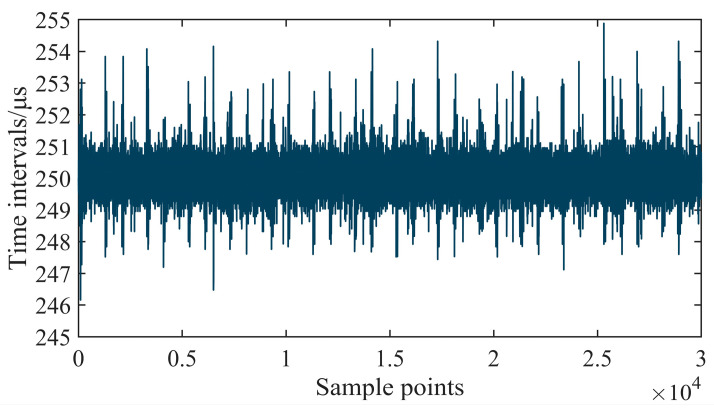
Test of EtherCAT real-time performance.

A large number of EtherCAT frames were captured during the motion control process. All of the frames sent from the master controller to the slaves were filtered out in Wireshark, and 30,000 adjacent frames were randomly selected from them. The time interval between every frame and the previous one was calculated based on the timestamp assigned by ALLBUS-TAP. As shown in Figure 24, the time intervals were about 250 μs, which corresponded to the EtherCAT communication cycle. The experimental results showed that the IgH EtherCAT master could send frames based on the preset cycle without packet loss. The maximum jitter of the cycle did not exceed 5 μs, indicating that the real-time performance of EtherCAT communication was very stable during motion control, which was conducive to improving the motion accuracy of the precision axes.

All of the experiments demonstrated that the IgH EtherCAT master can provide high-frequency and stable communication service in the process of motion control for precision axes. The errors of single-axis motion and dual-axis linkage are both smaller than the sub-micrometer level, and the motion control accuracy can meet the technical requirements of ultra-precision machine tools. However, this study only verified that the IgH EtherCAT master can control single or dual axes to perform ultra-precision motion with simple trajectories. An additional investigation is required to determine whether this method can achieve ultra-precision motion control in situations involving multi-axis and complex motion trajectories.

## 6. Conclusions

To test the feasibility of using IgH EtherCAT master for ultra-precision motion control of precision axes, this study developed a bus-type master controller based on the IgH EtherCAT master. In the master controller, an EtherCAT communication application and an interpolation application were designed, and a method of G-code data processing was proposed. These measures can effectively ensure accuracy and real-time performance for data processing and EtherCAT communication. An experimental platform was set up to carry out single-axis motion and dual-axis linkage control experiments with aerostatic linear axes. The experimental results showed that IgH could provide a stable 4 kHz EtherCAT communication frequency with a maximum jitter of less than 5 µs, which was beneficial to improving the motion control accuracy. Even in the presence of external error factors, the motion accuracy of single-axis positioning and dual-axis linkage control could reach the sub-micrometer level. This demonstrated that IgH EtherCAT master can fully achieve the ultra-precision control of precision axes in the case of executing simple motion trajectories on single-axis or dual-axis systems.

An additional investigation is needed to determine if this method is suitable for ultra-precision machine-tool processing with multi-axis motion and complex trajectories. If it proves to be capable, we can continue to enrich the functions of the master controller. This method will support researchers in transforming a PC into a bus-type ultra-precision motion controller based on the needs of machine tools. Thus, this approach will not only reduce the manufacturing costs of machine tools but also contribute to the functional expansion of ultra-precision machine tools.

## Figures and Tables

**Table 1:** Comparison of three measurements.

Measurement Number	Maximum Position Deviation (μm)	System Deviation (μm)	Average Deviation (μm)
1	1.166	1.621	1.326
2	0.142	0.167	0.108
3	0.085	0.111	0.102

**Table 2:** Evaluation of positioning accuracy.

Serial Number	Measurement Indicators	Measurement Results (μm)
1	Positioning accuracy (forward)	0.195
2	Positioning accuracy (reverse)	0.227
3	Positioning accuracy (entirety)	0.227
4	Repetitive positioning accuracy (forward)	0.118
5	Repetitive positioning accuracy (reverse)	0.196
6	Range of average deviation	0.077

## Data Availability

The original contributions presented in this study are included in the article. Further inquiries can be directed to the corresponding author.

## References

[1] Kong L., Cheung C., To S., Lee W., Du J., Zhang Z. (2008). A kinematics and experimental analysis of form error compensation in ultra-precision machining. Int. J. Mach. Tools Manuf..

[2] Chen L., Zheng J., Fan D., Chen N. (2023). Research on the High Precision Synchronous Control Method of the Fieldbus Control System. Machines.

[3] Cena G., Bertolotti I.C., Scanzio S., Valenzano A., Zunino C. (2012). Evaluation of EtherCAT distributed clock performance. IEEE Trans. Ind. Inform..

[4] Thomesse J.P. (2005). Fieldbus technology and industrial automation. IEEE Int. Conf. Emerg. Technol. Fact. Autom. ETFA.

[5] Vitturi S., Zunino C., Sauter T. (2019). Industrial Communication Systems and Their Future Challenges: Next-Generation Ethernet, IIoT, and 5G. Proc. IEEE.

[6] Wu X., Xie L. (2019). Performance evaluation of industrial Ethernet protocols for networked control application. Control. Eng. Pract..

[7] Paprocki M., Erwiński K. (2022). Synchronization of Electrical Drives via EtherCAT Fieldbus Communication Modules. Energies.

[8] Cao L.B., Wang T.Y., Jia S.H., Tian C., Tian Y. (2023). Innovation of EtherCAT adaptive synchronization control in embedded, C.N.C. Int. J. Commun. Syst..

[9] Grigoriev S.N., Martinov G.M. (2016). An ARM-based Multi-channel CNC Solution for Multi-tasking Turning and Milling Machines. Procedia CIRP.

[10] Pan C.T., Sun P.Y., Wang S.Y., Li H.J., Huang S.Y., Lin Z.C., Kuo C.H., Yen C.K., Wu C.N., Zheng J.L. (2020). Integration of multi-axis platform with synchronous motion-sensing and virtual reality imagery for the depth of immersion. Int. J. Adv. Manuf. Technol..

[11] You W., Kong M., Sun L., Diao Y. (2012). Control system design for heavy duty industrial robot. Ind. Rob..

[12] Hoffman A.J., Basson A.H. (2016). IEC 61131-3-based holonic control of a reconfigurable manufacturing subsystem. Int. J. Comput. Integr. Manuf..

[13] Cereia M., Scanzio S. A user space EtherCAT master architecture for hard real-time control systems. Proceedings of the 2012 IEEE 17th International Conference on Emerging Technologies & Factory Automation (ETFA 2012).

[14] Park S.M., Kim H.W., Kim H.J., Choi J.Y. (2020). Accuracy Improvement of Master-Slave Synchronization in EtherCAT Networks. IEEE Access.

[15] Yi H.C., Choi J.Y. Performance analysis of Linux-based EtherCAT DC synchronization. Proceedings of the 2015 IEEE International Conference on Advanced Intelligent Mechatronics (AIM).

[16] Delgado R., Kim S.Y., You B.J., Choi B.W. An EtherCAT-based real-time motion control system in mobile robot application. Proceedings of the 2016 13th International Conference on Ubiquitous Robots and Ambient Intelligence (URAI).

[17] Prytz G. A performance analysis of EtherCAT and PROFINET IRT. Proceedings of the 2008 IEEE International Conference on Emerging Technologies and Factory Automation.

[18] Rostan M., Stubbs J.E., Dzilno D. EtherCAT enabled advanced control architecture. Proceedings of the 2010 IEEE/SEMI Advanced Semiconductor Manufacturing Conference (ASMC).

[19] Zhong W.B., Luo X.C., Chang W.L., Cai Y.K., Ding F., Liu H.T., Sun Y.Z. (2020). Toolpath Interpolation and Smoothing for Computer Numerical Control Machining of Freeform Surfaces: A Review. Int. J. Autom. Comput..

[20] Barbalace A., Luchetta A., Manduchi G., Moro M., Soppelsa A., Taliercio C. (2007). Performance comparison of VxWorks, linux, RTAI and xenomai in a hard real-time application. IEEE Trans. Nucl. Sci..

[21] Pastorino R., Cosco F., Naets F., Desmet W., Cuadrado J. (2016). Hard real-time multibody simulations using ARM-based embedded systems. Multibody Syst. Dyn..

[22] Delgado R., You B., Han M., Choi B. (2019). Integration of ROS and RT tasks using message pipe mechanism on Xenomai for telepresence robot. Electron. Lett..

[23] Delgado R., You B.J., Choi B.W. (2019). Real-time control architecture based on Xenomai using ROS packages for a service robot. J. Syst. Softw..

[24] Dongwook K., Woojoong L., Park C. (2007). Kernel thread scheduling in real-time Linux for wearable computer. ETRI J..

